# A case report of primary upper urinary tract signet-ring cell carcinoma and literature review

**DOI:** 10.1186/s12894-020-00645-y

**Published:** 2020-06-26

**Authors:** Zhaohua Ye, Qiwu Mi, Daosheng Luo, Zhixiong Li, Jiexin Luo

**Affiliations:** Department of Urology, Dongguan People’s Hospital, Dongguan, 523000 China

**Keywords:** primary upper urinary tract signet ring cell carcinoma, calculi, percutaneous nephrolithotomy, radical nephroureterectomy

## Abstract

**Background:**

Upper tract urothelial carcinoma with pure non-urothelial histology is an exception but variants are present in ~ 25% of cases. Primary upper urinary tract signet -ring cell carcinoma is extremely rare.

**Case presentation:**

We report the case of a 65-year-old male diagnosed primary upper urinary tract signet-ring cell carcinoma while underwent percutaneous nephrolithotomy. Radical nephroureterectomy and adjuvant chemotherapy were performed sequentially. The patient is now recovering well with a regular follow-up for more than 1 year.

**Conclusions:**

The upper urinary tract malignancy often appears as a high grade, high stage tumor and has a uniformly poor prognosis, but a timely multimodal management can bring a good outcome.

## Background

Upper tract urothelial carcinoma (UTUC) with pure non-urothelial histology is an exception but variants are present in ~25% of cases [1, 2], including squamous cell carcinoma and adenocarcinoma. Among the cases mentioned above, upper urinary tract signet ring cell carcinoma (SRCC) is rare [3, 4]. The upper urinary tract malignancy often appears as a high grade, high stage tumor and has a uniformly poor prognosis [5]. Radical nephroureterectomy (RNU) with bladder cuff removal has been the standard treatment for UTUC [6]. But there is no standard perioperative therapeutic strategy exists for upper urinary tract SRCC because of their rarity.

## Case presentation

**Fig. 1:**
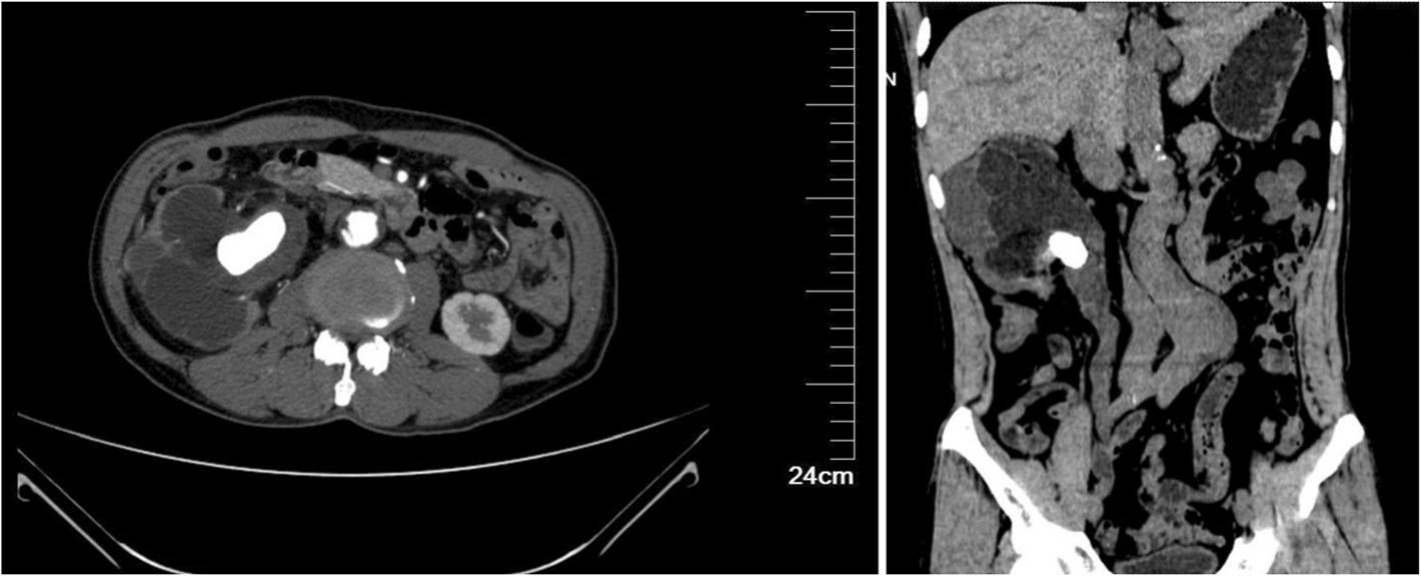
Abdominal CT demonstrated significant dilatation of the right renal pelvis and the right upper ureter with multiple calculi. The walls of the renal pelvis and the upper and mid-ureter were thickened with hyperdense soft tissue lesion

A 65-year-old male presented to our hospital with a 30 years history of right flank pain. His pain is a mild and intermittent dull pain, without radiation pain, gross hematuria, fever, urinary frequency or urgency and dysuria. The physical examination was unremarkable. Laboratory examinations showed Serum creatinine was 167umol/L (normal range 57-111umol/L), CEA, CA-199, and CA72-4 were normal. Abdominal CT demonstrated significant dilatation of the right renal pelvis and the right upper and mid-ureter with multiple calculi. The walls of the renal pelvis and the upper and mid-ureter were thickened with hyperdense soft tissue lesion. (Figure 1)

**Fig. 2:**
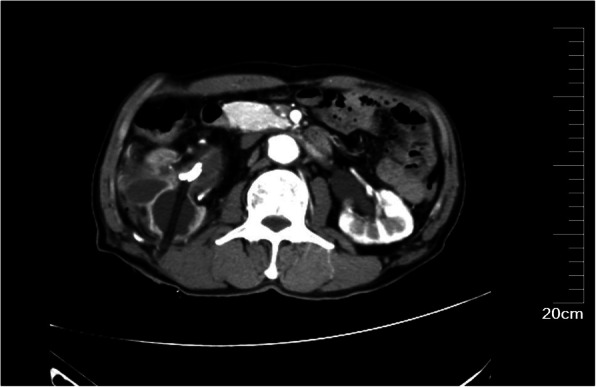
A 20-Fr Foley catheter was kept as a nephrostomy tube and the catheter balloon was pulled to compress the nephrostomy tract

**Fig. 3:**
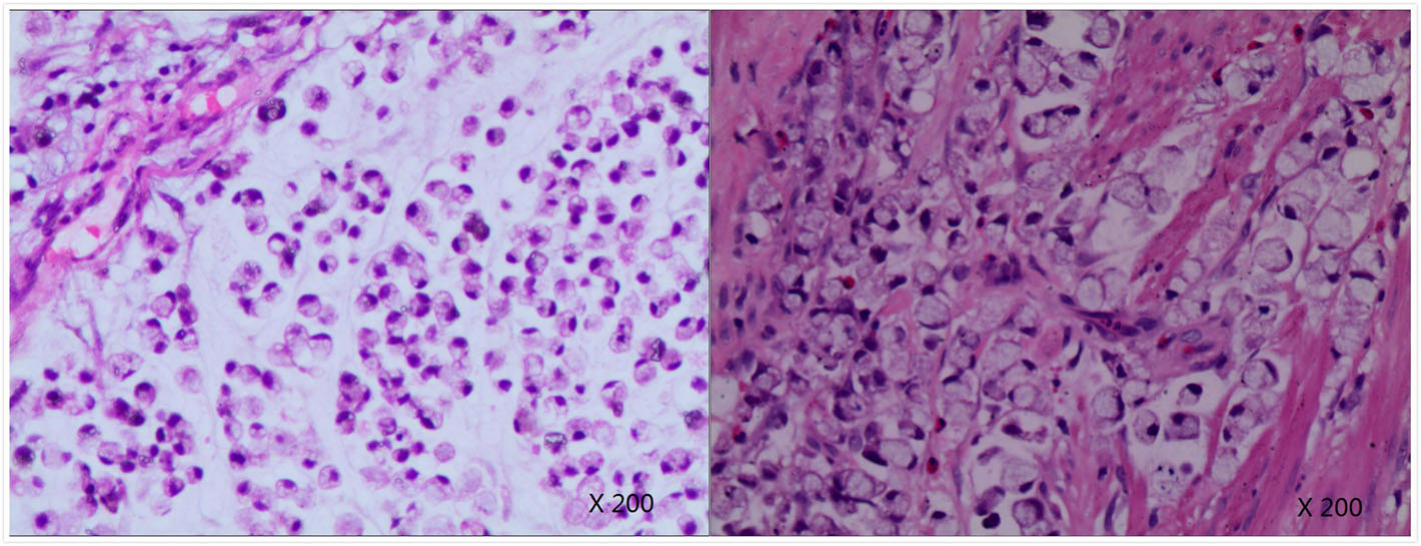
The histopathological examination showed the tumor cells containing intracellular mucin-filled vacuole displacing the hyperchromatic nucleus to one side suggestive of signet ring cell carcinoma

**Fig. 4:**
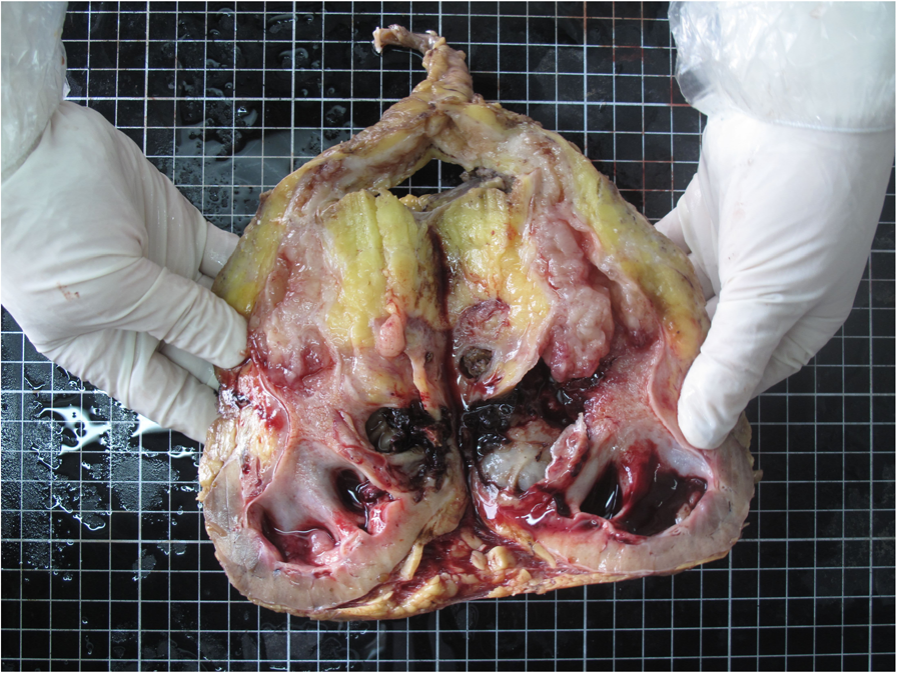
A right-sided radical nephroureterectomy with bladder cuff and nephrostomy tract sinus removal were performed

After three negative urinary cytology examinations, the patient underwent ureteroscopic examination, which showed middle ureteral wall was rough and stenosis with soft tissue mass, but biopsy specimens from this area were non-diagnostic. We tried to insert a double J stent to provide drainage but it failed. Then we recommended a right-sided radical nephroureterectomy but the families refused. They strongly required to the treatment of urinary calculi only. Then a right-side percutaneous nephrolithotomy (PCNL) was performed. Renal access was achieved using B-US guidance with 22-Fr Amplatz sheath. A large amount of white gelatinous material were found in the renal pelvis and taken out for histopathology examination. The calculi were either removed intact or fragmented using a pneumatic lithoclast. A 20-Fr Foley catheter was kept as a nephrostomy tube and the catheter balloon was injected with 10ml saline and pulled to compress the nephrostomy tract (Figure 2). The histopathological examination showed the tumor cells containing intracellular mucin-filled vacuole displacing the hyperchromatic nucleus to one side suggestive of signet ring cell carcinoma (Figure 3). Immunohistochemical examination showed: CK, CK7, CK20, CEA, CDX2 (+), Villin (small +), Vim, GATA3, P53 (-), Ki-67 (about 60% +).Post-operative PET/CT (18F-FDG8.6mCi as the tracer) showed there was no evidence of further primary malignancy or metastases. Therefore, we considered the tumor as primary upper urinary tract signet ring cell carcinoma. A right-sided RNU with bladder cuff and nephrostomy tract sinus removal were performed subsequently (Figure 4). Histopathology examination found signet-ring cell invaded the renal pelvis, upper ureter and the surrounding adipose tissue through the muscle, the focal nerve and vessels were also involved. Part of the renal pelvis epithelium was presented with obvious intestinal metaplasia and atypical hyperplasia, suggesting the tumor originated from the kidney. Six weeks after the surgery, he received 3 cycles chemotherapy (Gemcitabine 1000mg/m2 on days 1 and 8, every 21 days) as adjuvant therapy. 16 months postoperatively, clinical examinations and CT scans showed normal results without metastasis or localized recurrence.

## Discussion and conclusion

Urothelial carcinomas (UCs) are the fifth most common tumors [7], with the UTUC accounting for only 5-10% [8, 9]. Approximately 60% of UTUC are invasive at the time ofdiagnosis. The peak incidence of upper tract urothelial carcinoma occurs in the age from 70 to 90 years old, and the ratio of male to female is about 3:1 [10, 11]. Cases of histologically non-urothelial carcinoma are rare. Among these special cases, squamous cell carcinoma is most frequently reported, and there are variants such as micropapillary carcinoma, sarcomatoid carcinoma, SRCC, etc. Primary SRCC of the urinary tract is a relatively rare. Since primary SRCC of urinary bladder was first described by Saphir in 1955 [12], over 300 cases of urinary tract SRCC have been reported in the English literature, but the report about primary upper urinary tract SRCC is extremely rare. The first example of this variant had been documented by Ekfors and Nurmi in 1988 [13] and there are less than 10 cases so far. SRCC is most commonly found in the gastrointestinal tract. The pathogenesis of SRCC in the urothelium is not yet clear. Referring to the SRCC of urinary bladder, the current hypothesis holds that the signet-ring cell arising from the glandular metaplasia is caused by chronic inflammation or secondary to the stone stimulation [14–17] or from the totipotential urothelium by direct transition [18–20]. More scholars now support the first hypothesis. For this patient, a history of chronic stone stimulation did exist. Hematuria and flank pain could be the most common symptom at presentation, while a palpable abdominal mass indicates a late stage of the disease. However, it is difficult to distinguish stone from tumors only by symptoms and physical examination. Moreover, patients may be asymptomatic [21]. Although hematological oncology examinations are negative in most cases, including our case, abnormality could still be detected from CT scan. Fojecki G et al. concluded that CTU including corticomedullary phase (CMP) is the preferred imaging modality in the diagnostic workup of UTUC [22]. Histopathology is the golden standard for diagnosing UTUC, but there is no consensus which biopsy method is the best to achieve representative samples [23]. Urinary cytology and URS biopsy were negative neither for this patient. Two studies including 762 UTUC patients reported that the percentage of abnormal urothelial cells from preoperative cytology were found in the urine was only about 39% [24, 25]. Ureteroscopy is used to visualise the upper urinary tract and biopsy suspicious lesions. Flexible ureteroscopy is more useful with novel digital technology and biopsy device. However, Margolin et al. [26] obtained that the likelihood of missing invasion on URS biopsy was signifcantly increased when the diameter of biopsy fragments was smaller than 1 mm. That might be the reason for the false negative diagnosis of this case. This patient was finally diagnosed with upper urinary tract malignancy through PCNL. We had informed the risk and possible complications of PCNL, but the patient’s families insisted on kidney-sparing surgery. Admittedly, PCNL is not the most appropriate treatment for this patient, but we had taken the following strategies to avoid the spread of the tumor. Tract dilation was performed in a one-step method rather than a step-by-step method. The pressure and the flow rate of the irrigation fluid were under strict control. The total intraoperative time was 30 mins and the Foley catheter was used as a nephrostomy tube while the balloon was pulled tightly to compress the nephrostomy tract. The histopathological analysis of the specimens from renal pelvis revealed signet-ring cell components. Thus, a secondary SRCC or a rare variant of primary upper urinary tract SRCC was suspected. It is reported that histopathology is one of the method of identification. Stearns et al. described a case of a metastatic, undifferentiated gastric carcinoma, and stated that it grew mainly in adventitial or periureteral tissue without mucosal involvement [27]. Hes O et al. observed that metastatic cell usually grow in a dissociated manner [28]. In our case, part of the renal pelvis epithelium was presented with obvious intestinal metaplasia and atypical hyperplasia, suggesting the tumor originated from the kidney. Whether immunohistochemical analysis can be useful is still controversial. Singh J et al. described that immunohistochemical staining using CK7 and CK20 could also be helpful in evaluating the cancer’s primary origin [29] while Lendorf ME et al. observed that the immunoprofiles of primary urinary SRCC and SRCC arising from the gastrointestinal tract are overlap, including CK7, CK20, CEA, epithelial membrane antigen, CDX2, villin and E-cadherin [30]. Suh N et al. believed that it is indistinguishable based on histomorphology and immunohistochemistry [31]. Thus, upper gastrointestinal endoscopy and colonoscopy should be performed to rule out any eventual primary site. Since the patient refused invasive examination, a PET/CT was performed instead and it showed no evidence of further primary malignancy. Dong MJ et al. reported a case of the false negative ^18^F-FDG PET images of gastric SRCC with right adnexa metastasis. They observed that false negative ^18^F-FDG PET in malignant tumor may be correlated with the pathologic type, differentiation degree and the lesion size [32]. However, there is no case of symptoms of metastatic urinary tract SRCC appearing earlier than those of primary tumors and there is only one case of melanoma where symptoms of ureteral metastasis preceded the recognition of the primary neoplasm [33]. As we are writing, more than one year has passed since the diagnosis, and the patient is well without recurrence or metastasis. Although the follow-up time is not very long, in our opinion an occult primary tumor is unlikely. The RNU with bladder cuff and nephrostomy tract sinus removal and postoperative adjuvant chemotherapy (AC) was performed subsequently. Perioperative administration of chemotherapeutic agents has been explored for a long time. A phase 3 RCT from multi-institutional collaboration in the UK (POUT study), revealed that AC significantly improves disease-free survival and metastasis-free survival compared to surveillance and chemotherapy given at relapse in patients with UTUC [34]. Cobo-Dols M et al. [35] and El Ammari JE et al. [36] had respectively reported a success case of primary SRCC of bladder treated with radical cystectomy followed by systemic AC with cisplatin and gemcitabine. Considering there is no standard AC strategy for upper urinary tract SRCC, we referenced the chemotherapy for SRCC of urinary bladder. Meanwhile, the loss of renal function limited the use of platinum-based drugs, leading us to the administration of gemcitabine alone. According to the follow-up for more than one year after surgery, the prognosis of the patient is favorable. According to the TNM classification 2017 for urothelial carcinoma of the upper urinary tract, the pT3 category renal pelvis tumors are defined as the primary tumor invades beyond muscularis into peripelvic fat or renal parenchyma. Although there is no grading classification for upper urinary tract SRCC, it should be treated as high-grade disease. Thus, a long-term follow-up is still needed for this patient.

Patients with a long history of urinary calculi must be recognized the possibility of urinary tumors and regular workup should be done in order to diagnose the tumor in its early stage. Radiologic findings have a certain effect but non-specific. CTU including CMP is recommended. Histopathological examination is still the golden standard in the diagnosis of tumor occurs. Upper urinary tract SRCC is a rare tumor with a very poor prognosis that requires multimodal management. To plan the optimal therapeutic strategy, it is necessary to rule out a primary malignance outside the urinary tract. Treatment should be performed expeditiously given the aggressive nature of this disease and should include a combination of radical surgery and adjuvant chemotherapy. Prognosis is best predicted by pathologic grade and stage. Continued research is necessary to reveal the severity of this disease and standardize the diagnostic work-up and therapeutic strategy of upper urinary tract SRCC.

## Data Availability

For further details, the corresponding author can be contacted.

## Abbreviations

SRCC: Signet ring cell carcinoma
PCNL: Percutaneous nephrolithotomy
UTUC: Upper tract urothelial carcinoma
UCs: Urothelial carcinomas
RNU: Radical nephroureterectomy
CTU: Computed tomography urography
AC: Adjuvant chemotherapy
